# Transcriptome and Differential Expression Profiling Analysis of the Mechanism of Ca^2+^ Regulation in Peanut (*Arachis hypogaea*) Pod Development

**DOI:** 10.3389/fpls.2017.01609

**Published:** 2017-09-28

**Authors:** Sha Yang, Lin Li, Jialei Zhang, Yun Geng, Feng Guo, Jianguo Wang, Jingjing Meng, Na Sui, Shubo Wan, Xinguo Li

**Affiliations:** ^1^Biotechnology Research Center, Shandong Academy of Agricultural Sciences, Jinan, China; ^2^College of Agronomy, Hunan Agricultural University, Changsha, China; ^3^College of Life Sciences, Shandong Normal University, Jinan, China; ^4^Shandong Provincial Key Laboratory of Crop Genetic Improvement, Ecology and Physiology, Shandong Academy of Agricultural Sciences, Jinan, China

**Keywords:** calcium, peanut, pod development, RNA-Seq, differential expression analysis

## Abstract

Calcium not only serves as a necessary nutrient for plant growth but also acts as a ubiquitous central hub in a large number of signaling pathways. Free Ca^2+^ deficiency in the soil may cause early embryo abortion, which eventually led to abnormal development of peanut pod during the harvest season. To understand the mechanisms of Ca^2+^ regulation in pod development, transcriptome analysis of peanut gynophores and pods was performed by comparing the treatments between free Ca^2+^ sufficiency and free Ca^2+^ deficiency using Illumina HiSeq™ 2000. 9,903,082,800 nt bases are generated totally. After assembly, the average length of 102,819 unigenes is 999 nt, N50 is 1,782 nt. RNA-seq based gene expression profilings showed a large number of genes at the transcriptional level changed significantly between the aerial pegs and underground swelling pods under free Ca^2+^ sufficienct or deficiency treatments, respectively. Genes encoding key members of Ca^2+^ signaling transduction pathway, enzymes for hormone metabolism, cell division and growth, transcriptional factor as well as embryo development were highlighted. This information provides useful information for our further study. The results of digital gene expression (DGE) indicated that exogenous calcium might contribute to the development of peanut pod through its signal transduction pathway, meanwhile, promote the normal transition of the gynophores to the reproductive development.

## Introduction

Peanut (*Arachis hypogaea* L.) is an economic crop that contributes 20% to the global oil production and 11% of the protein supply per year (Chen et al., 2014). Aerial flowering and underground development of fruit are special genetic characteristics of peanuts, and the ecological environment of dark, moist and mechanical stimulation is essential conditions for pod development. So, the ovule-carrying pegs (gynophores) must elongate to bury the fertilized ovule into the soil in order to survive and reproduce (Arya et al., 2016). When gynophores are buried into the soil at 2–8 cm deep, the ovule swells to provide room for the embryo to grow. During this complex period, the peanut can directly absorb moisture, calcium, and other inorganic salts from the soil to maintain its reproductive development (Beringer and Taha, 1976). Calcium-deficient soil may result in the termination of pods expansion and eventually lead to embryo abortion. By contrast, peanuts will produce filled pods when sufficiently supplied with calcium (Jain et al., 2011). Therefore, the development of peanut pod is extremely sensitive to calcium, and free-Ca^2+^ deficiency in the field will greatly reduce the yield of peanut.

Ca^2+^, a universal second messenger, plays an important role in plant growth and development, including cell division and apoptosis, polarity formation, differentiation and senescence (Hepler, 2005; Zhang et al., 2017). In our previous study, we reported that free-Ca^2+^ deficiency results in peanut dwarfism, and Ca^2+^/CaM signal transduction pathway was involved in the turnover of PSII reaction center components to alleviate the damage of photoinhibition to PSII (Yang et al., 2013, 2015). However, little was known about the regulation of calcium on the development of peanut pods over the last century. With the development of molecular biological techniques, the transcriptome and proteomics benefited the peanut genomics research, and was applied in the seed development. Recently, several studies have compared genes and proteins between the aerial and underground peanut pods (Zhu et al., 2013). Chen et al. (2013) speculated that categories in the photosynthetic pathway were altered greatly in aerial gynophores, while the growth regulators such as IAA, ABA and kinetin may be the key factors contributing to the swelling of the subterranean pods. Other studies have explored changes in the gene expression at the different developmental stages of the gynophores. Xia et al. (2013) assembled 13 million short sequences into 72,527 unigenes to study peanut gynophores in three developmental stages. The results showed that numerous enzymes involved in plant hormone biosynthesis and signaling pathway, as well as light signaling, changed significantly when the ovary began to enlarge. These candidate genes and proteins have been identified to understand the regulation mechanisms that control the development of peanut pods and provide valuable resource to illustrate the specific mechanism of calcium signaling on pod development.

Digital gene expression (DGE) profiling, by comparing reads with the reference genome, can comprehensively and rapidly detect the specific gene expression of a particular species and distinguish the differences in the expression of poorly expressed genes (Glazinska et al., 2017). However, only reference transcripts from wild peanuts, not those of the cultivated peanut, have been published. Therefore, transcriptome profilings obtained from a mixture of all samples tested in our study will supply the data for the reference genome. Transcriptome sequencing can be used to sequence all mRNAs transcribed by eukaryotes, specific tissues, or cells in a particular state, and also analyze the structure of the genes and new transcripts produced (Sathiyanathan et al., 2017). This method has been widely applied in *Arabidopsis* and other model plants, e.g., rice and soybean, and has gradually become a useful tool in biological research.

In this study, RNA-Seq and digital gene profiling were combined to investigate and compare differentially expressed genes (DEGs) between aerial gynophores and underground pods grown under free-Ca^2+^-sufficient or deficient Our objectives were to: (1) compare the DEGs of aerial gynophores between free Ca^2+^ sufficiency and free Ca^2+^ deficiency treatments, (2) identify candidate genes related to pod swelling affected by calcium, (3) find out the main regulatory pathways of calcium to promote pod development.

## Materials and methods

### Plant materials and treatments

The *Arachis hypogaea* “XiangHua 2008,” a peanut cultivar from the Hunan Agriculture University, was used as material. The red soil overburden including exchangeable calcium 0.74 cmol(1/2 Ca^2+^)/kg in Gengyuan practice base, Hunan Province of China, was used for the free Ca^2+^ deficient treatment. The soil fertilized with 100 kg plaster (CaO) per 667 m^2^ was used as free Ca^2+^ sufficiency treatment. CaO was applied before sowing.

### The method of sampling

In peanut production, 15 days after pegging (DAP) was confirmed to be the most sensitive period for the free Ca^2+^ content in the soil. In this study, gynophores under free Ca^2+^-deficient treatment 1 (GD1), free Ca^2+^-deficient treatment 2 (GD2), free Ca^2+^-sufficient treatment 1 (GS1), free Ca^2+^-sufficient treatment 2 (GS2), as well as pods under free Ca^2+^-deficient treatment 1 (PD1), free Ca^2+^-deficient treatment 2 (PD2), free Ca^2+^-sufficient treatment 1 (PS1), and free Ca^2+^-sufficient treatment 2 (PS2), were harvested, frozen in liquid nitrogen and then stored at −70°C. The 8 samples were used to detect global changes in gene expression. Two biological replicates were used in this study. The mixed samples were used in transcriptome sequencing.

### RNA extraction and sequencing

Total RNA was isolated from the harvested materials using the total RNA extraction Kit (TaKaRa, Inc., Dalian, China) according to the manufacturer's instructions. Agilent 2100 and NanoDrop were used to detect RNA quality and purity. Only high-quality RNA samples were chosen for RNA-seq analyses. Beads with Oligo (dT) were used to enrich for mRNA and the RNA was fragmented into short fragments. First-strand cDNA was synthesized using random hexamer primers and buffer, while dNTPs, RNaseH and DNA polymerase I were added to synthesize the second strand (Wang et al., 2009). After purification, the short fragments were then connected using sequencing adapters. Finally, the library was sequenced from directions on Illumina HiSeq™ 2000 System (Illumina, San Diego, CA). Data analysis and base calling were achieved by applying the Illumina instrument software. Whole dataset has been deposited in the NCBI Sequence Read Archive with accession number SRX1795063.

### Digital expression profile analysis

Differential expression profiling was conducted among the gynophores and pods under free- Ca^2+^-deficient or sufficient treatments. Prior to further analysis, raw data should be filtered to decrease data noise and obtain clean data (Cock et al., 2010). Gene expression level was quantified by a software package RNASeq by Expectation Maximization (*RSEM*). *RSEM* computes maximum likelihood abundance estimates using the Expectation-Maximization (EM) algorithm for its statistical model, including the modeling of paired-end (PE) and variable-length reads, fragment length distributions, and quality scores, to determine which transcripts are isoforms of the same gene (Li and Dewey, 2011). Fragments per kilobase of exon per million fragments mapped (FPKM) method was used to calculate the expression level as follows: FPKM = [10^6^C/(NL/10^3^)], where FPKM is the expression of gene A, C is the number of fragments uniquely aligned to gene A, N is the total number of fragments uniquely aligned to all genes, and L is the number of bases on gene A (Ning et al., 2013). The FPKM method can eliminate the influence of different gene lengths and sequencing discrepancy on the calculation of gene expression. The intensity values of each sample were further transformed on log2-scale and used to analyze differential expression. The probe sets with *P* < 0.01 in at least one of the comparisons were considered as DEGs for further analysis. The WEGO software was used to obtain gene ontology (GO) annotations for the DEGs, while pathway analyses were used to analyze KEGG and BLASTX (*E* < 0.00001) against the NCBI Nr database (Ye et al., 2006).

The contents of endogenous hormone indole acetic acid (IAA) and gibberellin (GA_3_) were determined by enzyme linked immunosorbent assay (ELISA), which was performed as a commercial service in China agricultural university.

### Quantitative real time PCR (qRT-PCR) analysis

Gynophores and pod samples under the same treatments as those in RNA-seq were analyzed using qRT-PCR. cDNAs were reverse transcribed using the PrimeScript™ first-strand cDNA synthesis kit (K1622 Thermo scientific). The PCR was amplified using SYBR Premix Ex Taq™ following the manufacturer's instructions (TaKaRa, Inc., Dalian, China) with the qRT-PCR amplification instrument (ABI 7500, USA). The target gene primers (Table S3) were designed to detect the sample mRNA. Tua5-F and Tua5-R were used as controls to normalize the expression data (Yang et al., 2013). The relative gene expression was calculated using the 2^−ΔΔCT^ method as described by Livak and Schmittgen (2001). For log_2_-transformed FPKM values, the maximum expression level of each selected gene was considered to be 100, and the expression levels of the other genes were transformed accordingly (Zhang et al., 2016).

## Results

### Transcriptome sequencing and data analysis

Ca^2+^ deficiency in soil induces early embryo abortion in peanuts, and this process produces empty pods. This phenomenon was verified by the full pod number and dry weight of pods (Table 1). In our study, we attempted to reveal the molecular mechanism of the effect of free calcium on the development of peanut pods using a RNA-seq approach. A total of 99,030,828 clean reads with an average length of 90 bp, which corresponded to approximately 8.91 gigabase pairs (Gb) of raw data, were obtained. An overview of the sequencing and assembly is outlined in Table S1. 141,819 contigs with an average length of 391 bp were produced. Among these contigs, the length of 81,881 (57.73%) from 100 to 200 bp, 18,847 (13.28%) from 200 to 300 bp, 9256 (6.52%) from 300 to 400 bp, 5633 (3.97%) from 400 to 500 bp, and 26,202 (18.47%) were more than 500 bp in length. The results of the assembly showed 102,819 unigenes with a total length of 102.72 Mb and an average of 999 bp. Of these, the length of 46,924 (45.63%) were from 100 to 500 bp, 17,974 (17.48%) from 500 to 1,000 bp, 12,891 (12.53%) from 1,000 to 1,500 bp, 10,011 (9.73%) from 1,500 to 2,000 bp, and 15,019 (14.60%) were more than 2,000 bp (Figure S1). The length of assembled sequences is a criterion for successful assembly. We calculated the length distribution of contigs and unigenes, and the results showed that the Illumina sequencing solution was reproducible and reliable (Figure S1).

**Table 1:** The effects of calcium treatment on peanut phenotype.

**Treatment**	**Branch number**	**Dry weight of root and shem (g)**	**Full pod number**	**Dry weight of pods (g)**
CK	11.67 ± 1.15a	9.82 ± 1.21a	9.33 ± 0.58a	23.65 ± 1.57a
NC_12_	16.67 ± 1.15b	15.25 ± 1.37b	16.67 ± 1.15b	33.65 ± 1.68b

*a, b means significant difference compared with CK and calcium-treated plants (NC12) using Student's t-test at P < 0.05*.

### Unigene function annotation

**Figure 1:**
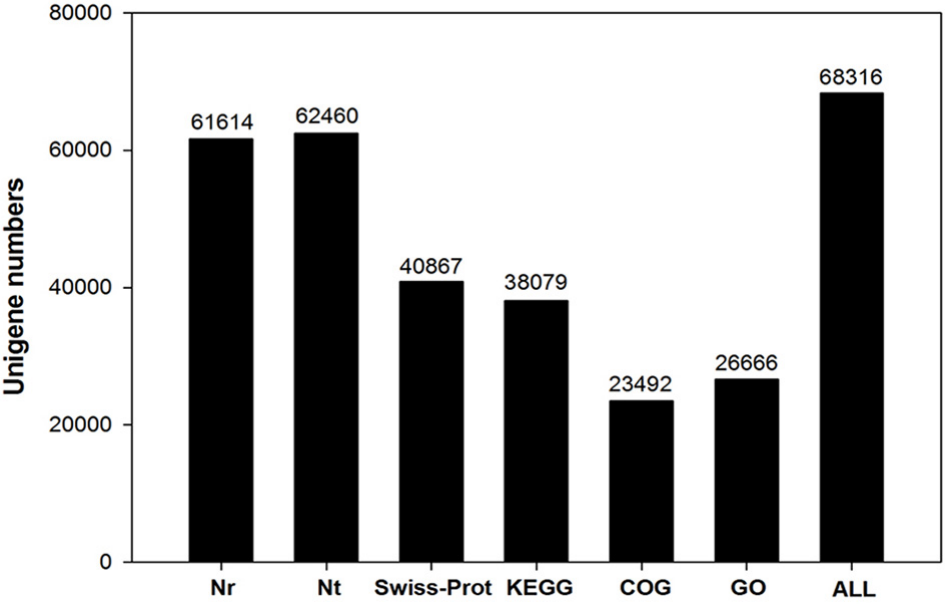
The unigene number annotated in six public database searched.

**Figure 2:**
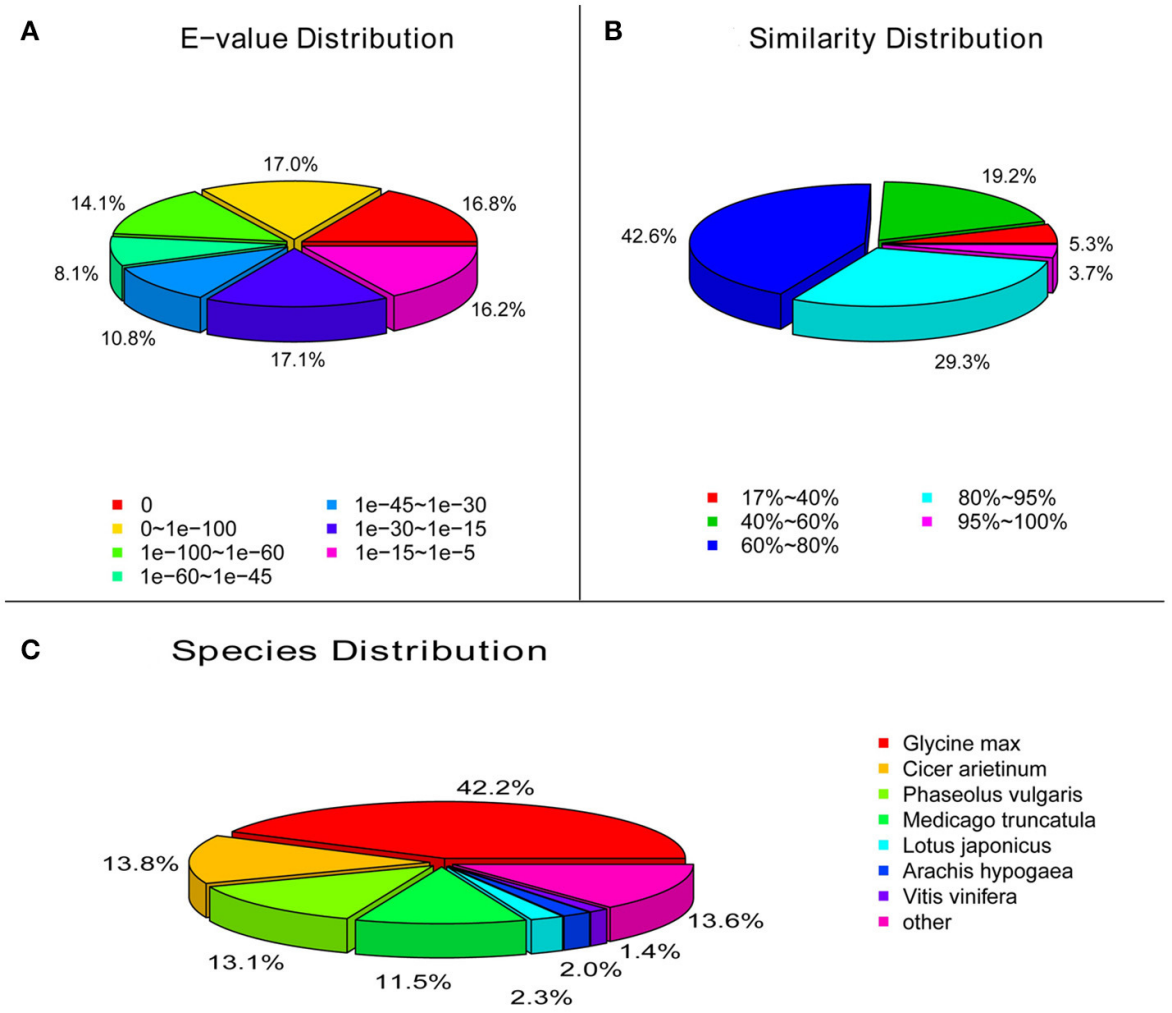
Characteristics of similarity search of unigenes against Nr database. **(A)**, *E*-value of BLAST hits for each unigene. **(B)**, Similarity distribution of the top BLAST hits for each unigene. **(C)**, Species distribution of the top BLAST hits for each unigene in Nr database.

Functional annotation of unigenes gives protein functional annotation, COG functional annotation and Gene Ontology (GO) functional annotation. Unigenes were annotated with the databases of NR, NT, Swiss-Prot, KEGG, COG, and GO. Then the number of annotated unigenes was counted with each database (Figure 1). Analysis of species distribution showed that 42.2% of the unigenes were homologous with the sequence of *Glycine max*, and 13.8, 13.1 and 11.5% of the unigenes with the sequence of *Cicer arietinum, Phaseolus vulgaris*, and *Medicago truncatula*, respectively (Figure 2).

We use Blast2GO program with NR annotation to obtain the GO annotation of the unigenes. GO has three ontologies: molecular function, cellular component and biological process. Every GO-term belongs to a type of ontology (Conesa et al., 2005). Figure S2 showed that the sequences were categorized into 55 functional groups according to the sequence homologies. We mapped the unigenes to the COG database to further predict the possible functions and statistics. In the 25 COG categories, general function prediction (7,524, 32.03%) represented the largest group, followed by transcription (4,202, 17.89%), and replication, recombination, and repair (4,128, 17.57%) (Figure S3).

A total of 14,985 sequences, containing 18,215 potential EST-SSRs, were detected with software MicroSAtellite (MISA). The quantity statistics of SSR classification showed that tri-nucleotide (40.86%) was the most abundant type of repeat motif, followed by di-nucleotide (29.94%), mononucleotide (21.61%), hexa-nucleotide (2.67%), quad-nucleotide (2.53%), and penta-nucleotide (2.38%) repeat units (Table S2). Within the cSSRs detected, AG/CT represented the dominant type, followed by AAG/CTT, ATC/ATG, and AAT/ATT (Figure S4).

### Digital gene expression (DGE) profiling

Eight DGE libraries were created from four independent biological samples using gynophores and pods with free Ca^2+^-deficient or -sufficient treatments as described in the method section. The clean reads (in millions) obtained from the different libraries were as follows: GD1, 14.91; GD2, 15.59; GS1, 14.54; GS2, 14.34; PD1, 16.02; PD2, 16.67; PS1, 14.19; and PS2, 12.84. The saturation analysis showed that when the number of reads reached a certain amount, the growth curve of detected genes tended to flatten in all libraries, indicating that the sequencing was saturated for gene identification (Figure S5).

**Figure 3:**
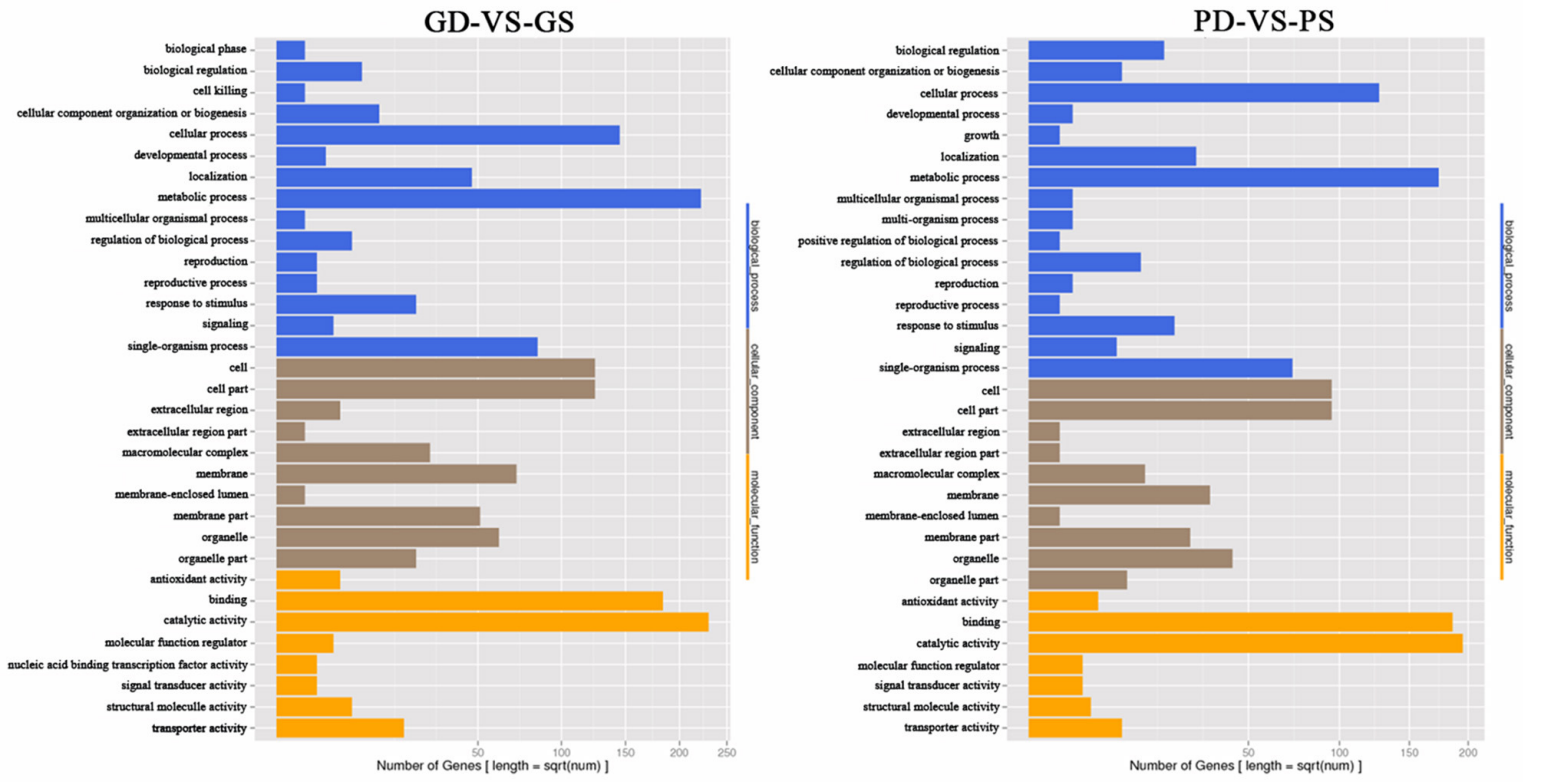
GO annotation of the DEGs from the two pairwise comparisons (GD/GS, PD/PS).

GO annotation of the DEGs from two pairwise comparisons (GD/GS and PD/PS) was used to classify genes into different sub-categories belonging to the three main GO categories. In the biological process category, we identified three abundant sub-categories across both comparisons, namely, metabolic process, cellular process, and single organism process. Cell, cell part, and membrane were the main sub-categories identified in the cellular component category, while catalytic activity and binding dominated the molecular function category (Figure 3).

KEGG enrichment analysis allowed the mapping of DEGs to different pathways. The top 20 pathways in each pairwise comparison (mentioned above) are shown in Figure S6. DEGs were mainly enriched in photosynthesis, phagosome, glycolysis/gluconeogenesis, isoflavonoid biosynthesis, glycosylphosphatidylinositol (GPI)-anchor biosynthesis, carbon metabolism, and ABC transporters in GD-VD-GS. Meanwhile, circadian rhythm-plant, plant hormone signal transduction, phagosome, isoflavonoid biosynthesis, glycolysis/gluconeogenesis, fatty acid metabolism, and biosynthesis of unsaturated fatty acids were mainly enriched in PD-VD-PS.

### Analysis and qRT-PCR validation of DGE results

**Figure 4:**
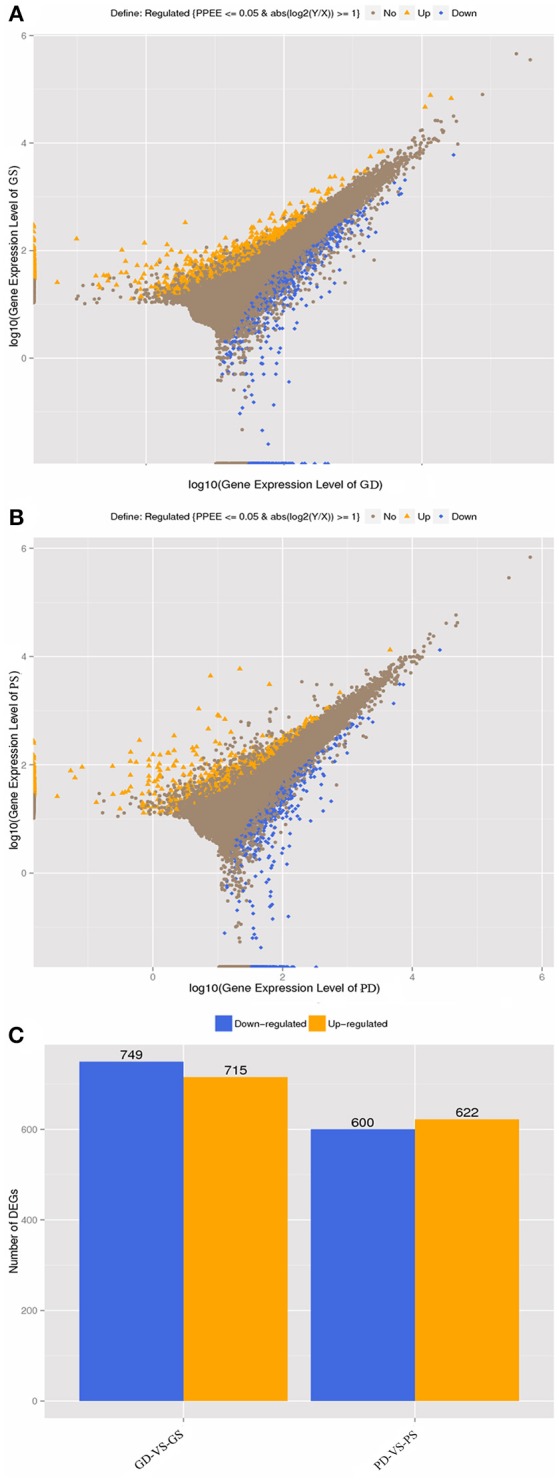
Differentially expressed genes in GD, GS, PD, and PS. **(A)**, gene expression level (GD-vs.-GS). **(B)**, gene expression level (PD-vs.-PS). **(C)**, Statistic of differentially expressed genes between GD-vs.-GS and PD-vs.-PS, respectively.

FPKM method was used to calculate the gene expression level. The reference transcripts of a progenitor of cultivated peanut (*Arachis ipaensis*) and our transcriptome data were used to generate an integrated reference library. For result list of each control-case pair, we draw scatter plots of all expressed genes, different color presents up-regulated, down-regulated or non-regulated genes. The total number of DEGs for each comparison was observed, and a histogram was drawn to show the number of significantly up-regulated or down-regulated genes in each control–case pair (Figure 4).

**Figure 5:**
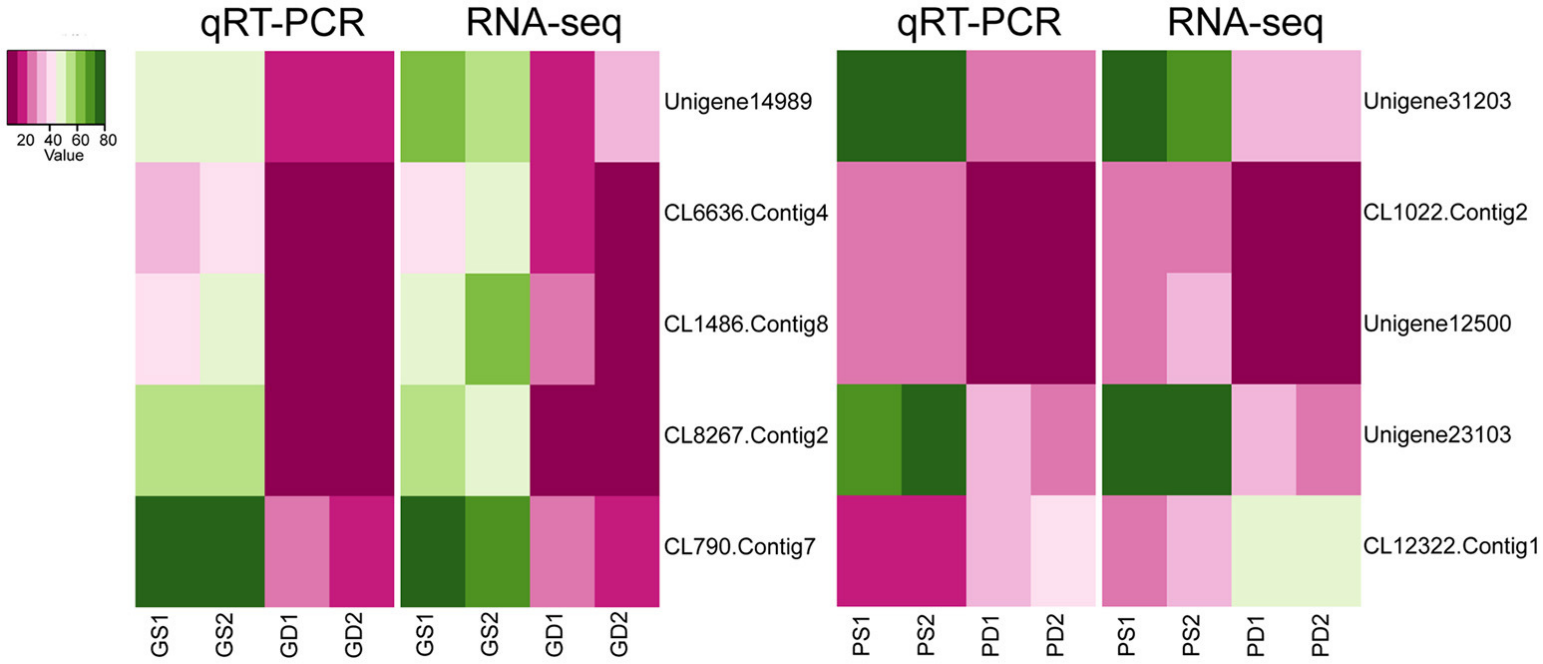
qRT-PCR verification of expression of selected genes.

In order to confirm the transcriptome sequencing results, 10 DEGs in GS, GD, PS, and PD were selected randomly for qRT-PCR to analyze the expression levels. The selected genes participate in calcium signal transduction and phytohormone biosynthesis pathway, material biosynthesis, transcription factor and transporter. The consistency of trends between qRT-PCR and RNA-seq data indicated the credibility of the RNA-seq data (Figure 5).

### Comparative analysis of genes between GD and GS

More than 20 unigenes annotated as transcription factors including NAC, WRKY, bHLH, and ethylene-responsive transcription factors were regulated in GS, while M1/AGAMOUS/DEFICIENS/SRF (MADS) transcription factor family were down-regulated in GS compared with GD. Contrary to the DEGs in pods, no significant difference in calcium-related genes between GD and GS was found, while the expression of some calcium-binding proteins (CBPs), and calcium-binding transcription factors were up-regulated in GS (Table S4).

The expression pattern of lipoxygenases (*LOX*) genes showed higher expression level in GS and the expression levels of 6 *LOX* genes were more than 2 times higher in GS than that in GD. Genes related to hormone response were screened out by GO analysis. Several DEGs involved in biosynthesis process and signaling pathway of gibberellin (GA), auxin, ABA, and brassinosteroid were up-regulated in GS at this stage. e. Numerous transporter-related genes were screened from DEGs based on GO analysis. Several energy- and substance-related transporter genes, including sugar transporter, carbohydrate transmembrane transporter, lipid transporter, protein transmembrane transporter, inositol transporter, zinc transporter, sulfate transporter, and nucleobase transporter were all up-regulated in GS. Meanwhile MFS transporter, ABC transporter and myo-inositol transporter had both up-regulated and down-regulated. Only the peptide/histidine transporter was down-regulated in GS (Table S4).

### Differentially expressed genes between PD and PS

We identified the DEGs between PD and PS considering that calcium plays an important role in the development of peanut pods. Unigenes encoding proteins involved in calcium signaling transduction pathways such as calcium-dependent protein kinases (CDPKs), calcium-binding protein (CBP) and calmodulin (CaM)-binding protein were up-regulated under free Ca^2+^-sufficient conditions. In particular, the expression of Ca^2+^-related protein mitogen-activated protein kinase kinase kinase (MAPKKK) was also higher in PS than that in PD (Table S5).

**Figure 6:**
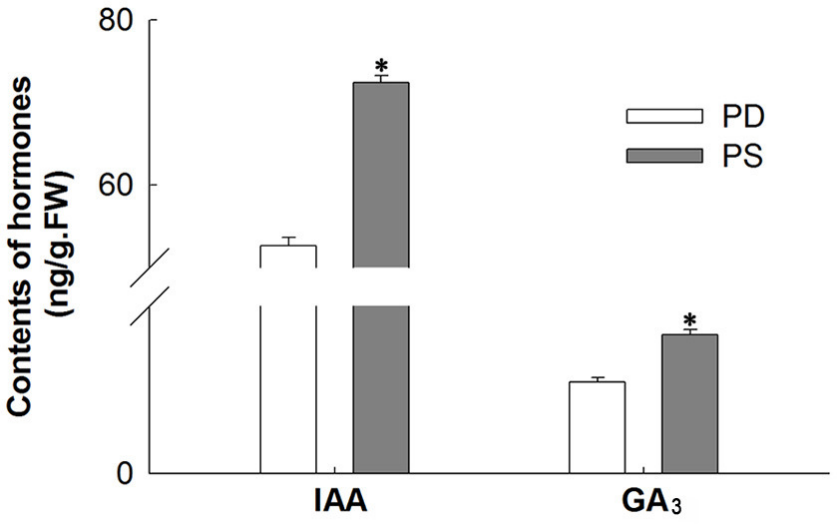
Compare IAA and GA_3_ contents between PS and PD. ^*^Significant difference compared PD with PS using Student's *t*-test at *P* < 0.05.

Two categories of plant hormone-related genes (auxin and GA) showed significantly different expression between PD and PS. The auxin-related genes including 7 auxin response factors (ARFs) and 1 indole-3-acetic acid-amido synthetase were up-regulated in PS. Two selected DEGs, namely, GA 20-oxidase and GA receptor GID1, were up-regulated, while GA 2-oxidase, which is one of the key enzymes that catalyzes the inactivation of biological GA, was down-regulated in PS. Meanwhile, the ent-kaurenoic acid oxidase (KAO), an important enzyme in GA biosynthesis (Yamaguchi and Kamiya, 2000; Paparelli et al., 2013), was up-regulated under free Ca^2+^-sufficient conditions. These results were in agreement with the high contents of IAA and GA_3_ in PS (Figure 6). Thus, our results indicated that the up-regulated calcium-related genes increased GA level, which may play an important role in the early embryo development.

Genes encoding storage protein oleosin and some synthetases, such as tetrahydrocannabinolic acid synthase, long chain acyl-CoA synthetase, and callose synthase, exhibited higher level (5–8-folds) under free-Ca^2+^-sufficient conditions. In addition, eight lipoxygenase were also up-regulated in PS. Previous studies indicated that lipoxygenase is the first key enzyme in the synthesis pathway of jasmonic acid, which plays an important role in the germination, growth, and stress resistance of plant seeds (Rahimi et al., 2016). GIGANTEA, a gene involved in circadian clock and phytochrome signaling (Cha et al., 2017), was down-regulated in PS (Table S5). This phenomenon may be due to the fact that this light response gene cannot play a regulatory role in the subterranean areas, because darkness is also a prerequisite for the normal development of peanut pods.

## Discussion

Early embryonic development is an important period for the formation of peanut yield and quality. Accumulating evidence illustrated that this complex process is regulated by light, endogenous hormones and environment stimuli. Ca^2+^ deficiency in this process leads to early embryo abortion in peanut, resulting in unfilled pod and reduced peanut yield. This phenomenon is a long-term concern in the acidic red-yellow soil in southern China (Zhang et al., 2007). *AhCYP707A4*A has been identified to regulate embryo abortion induced by Ca^2+^ deficiency using SSH cDNA libraries associated with library lift (SSHaLL) (Chen et al., 2015). However, SSHaLL is a less sensitive method that cannot detect the gene expression of whole species. Thus, this process provides limited information, and the underlying mechanism remains unclear.

*De novo* transcriptome sequencing facilitates the complete and rapid acquisition of almost all transcripts of a particular organ or tissue of a species based on high-throughput technologies. In this study, we characterized and compared gene expression profiles in gynophores and pods under free Ca^2+^-sufficient or free Ca^2+^-deficient treatment to identify candidate genes related to Ca^2+^ regulation on pod development. Our project was sequenced on the platform of Illumina Hiseq 2000. A total of 9,903,082,800 nt bases were generated. For function annotation analysis, we obtained 61,614, 62,460, 40,867, 38,079, 23,492, and 26,666 unigenes annotated to the NR, NT, Swiss-Prot, KEGG, COG, GO database, respectively (Figure 1). The total number of SSR was 18,215 (Figure S4). These unigenes and the reference transcripts of *Arachis ipaensis* can serve as reference sequences to determine the regulation pathway of calcium on peanut pod development.

In recent years, with widespread application of sequencing technology, transcriptome and proteomics provide views to explore the mechanism of the development of aerial peg and swelling of underground pod (Chen et al., 2013). The mechanisms of calcium on the aerial and underground part of peanut have not been reported yet. In our study, MADS-box family was significantly up-regulated in gynophores under calcium-deficient condition (Table S4). MADS box transcription factors are mainly known as key regulators of seed and flower development in *Arabidopsis*. Overexpression of *OsMADS45* induces early flowering and premature senility of gynophores (Wang et al., 2013; Yu et al., 2017). Hence, calcium deficiency probably prevented the swelling of the gynophores to form a pod and resulted in abortion. LOXs, which are key genes that play important roles in the production of growth regulators and the mobilization of stored lipids during seed germination, exhibited differential transcription levels in GS and GD (Gao et al., 2011). The up-regulated expression of LOXs in GS is likely to provide sufficient nutrient preparation for pod development. Combined with the high expression of several energy- and substance-related transporter genes, which play critical roles in nutrient uptake and signal transduction (Leem et al., 2016), exogenous calcium probably promotes nutrient storage in the aerial gynophores, ascertaining the early development of pod.

Members of the Ca^2+^ signaling pathway, including CaM-binding protein, CDPK, and MAPKKK, were found to be highly expressed in PS (Table S5). CaM has no inherent catalytic activity, and requires the modulation of its downstream binding proteins to function (Leba et al., 2012; Poovaiah et al., 2013). Thus, CaM has an effect on transmitting the calcium signals to downstream proteins and regulating plant growth and development. CDPKs, which are calcium sensors, are also implicated in the growth of plants, such as pollen development and gravitropism, and their response to environment stresses, such as pathogens (Ormancey et al., 2017). The MAPK cascade consists of interlinked MAPK, MAPKK, and MAPKKK and such cascades play important roles in signal transduction of plant hormone, biotic stress and abiotic stress (Wang et al., 2017). By contrast, in aerial gynophores, no significant difference was found in the Ca^2+^ sensors between GD and GS, and only some CBPs and calcium-binding transcription factors were up-regulated in GS. It is probably interesting to investigate that calcium signal transduction pathway may participate in regulating the development of peanut pod.

**Figure 7:**
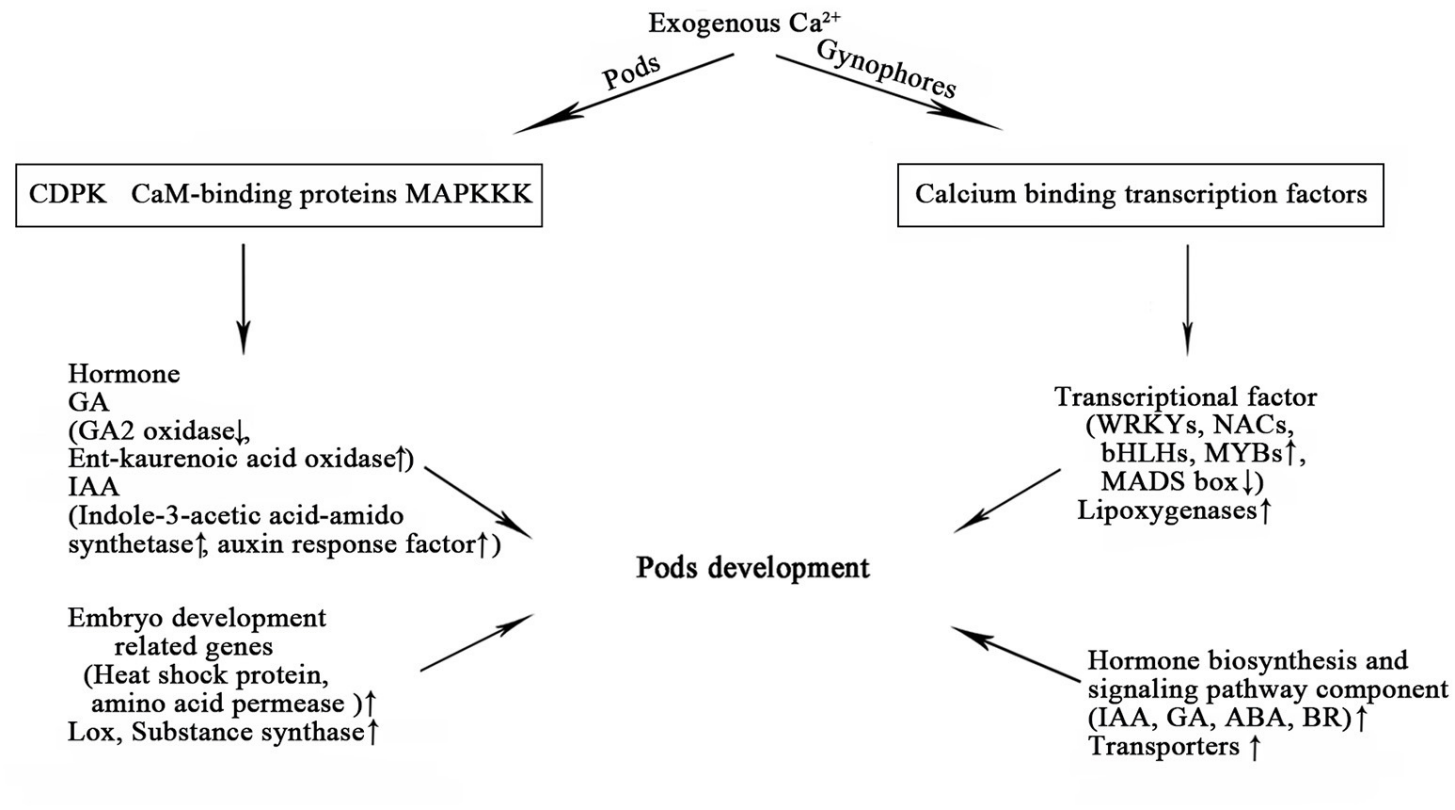
Identified DEGs from gynophores and pods under free Ca^2+^ sufficient treatment and their functions during early pod development.

Several studies have indicated that plant hormones, such as auxin, kinetin, ABA, and ethylene, can temporally and spatially coordinate the elongation of gynophore and swelling of pod (Jacobs, 1951; Ziv and Zamski, 1975; Shlamovitz et al., 1995). In this study, several enzymes in the synthesis pathway of GA were screened out to determine significant DEGs in pods between Ca^2+^-sufficient and Ca^2+^-deficient conditions, which were not found in gynophores. Low expression of GA_2_-oxidase and high expression of ent-kaurenoic acid oxidase under sufficient exogenous calcium eventually led to high contents of GA (Figure 6). GA is reported to be required to enhance seed germination, and the increase in the synthesis of active GA contributes to the promotion of seed germination (Song et al., 2011). Therefore, calcium may promote peanut seed germination and development through GA biosynthesis pathway. This novel discovery has not been reported in previous studies. Auxin plays a critical role in plant organ development. Genes encoding auxin response factors (ARF) and indole-3-acetic acid-amido synthetase exhibited increased changes in the PS (Table S5). ARF is a transcription factor that activates or represses the expression of auxin response genes. In *Arabidopsis*, ARF5 can promote the development of cotyledon by recruiting DRN expression, and to a great extent, ABA function in seed germination depends on the TIR1/AFB-AUX/IAA-ARF–mediated auxin signaling pathway (Liu et al., 2013). Therefore, the mechanism by which auxin-related genes identified in our study respond to calcium signaling during peanut seed development needs further research. The change in these hormones under sufficient calcium condition is beneficial for the normal development of peanut pod. Thus, the results elucidate a crucial mechanistic role of calcium on pod development. On the one hand, calcium enhanced the storage of aerial nutrients. On the other hand, it activated calcium signaling pathways and hormone-related genes in embryonic development (Figure 7). All these elements ensure the normal development of pods.

## Conclusion

In this study, we performed a comparative transcriptome and differential expression analysis of peanut gynophores and pods under free Ca^2+^ sufficient and deficient conditions, respectively. Transcription factors including WRKY, NAC, bHLH, and MADS-box transcription factors were found up-regulated in GS, while MADS transcription factor family were down-regulated in GS compared to GD. Genes involved in plant hormone biosynthesis and transporters were found to be differentially expressed in the GS and GD. Different from the aerial part of peanut, genes involved in calcium signaling transduction pathways were identified up-regulated in pods when free Ca^2+^ was sufficient. In addition, hormone and embryo-related genes also showed differentially expressed between PD and PS. To our best knowledge, we firstly provided mechanism of exogenous calcium on the aerial and underground parts of peanut at the early stage of seed development.

## Author contributions

SW and XL designed the experiment and drafted the manuscript. SY and LL carried out most of the experiment, analyzed the transcriptome and digital gene expression data, wrote the methods part of the manuscript. JZ, FG, and JM performed experiments, JW, NS, and YG grew the plants and prepared the samples. All authors read and approved the final manuscript.

### Conflict of interest statement

The authors declare that the research was conducted in the absence of any commercial or financial relationships that could be construed as a potential conflict of interest.

## Supplementary material

Figure S1 The length distribution of contigs and unigenes.

Figure S2 GO classification analysis of unigenes in All-unigene. GO functions is showed in X-axis. The right Y-axis shows the number of genes which have the GO function, and the left Y-axis shows the percentage.

Figure S3 Clusters of orthologous groups (COG) classification of Unigenes in All-Unigene.

Figure S4 Quantity statistics of SSR classification. The X-axis is the repeat times of repeat units. The Y-axis is the number of SSRs.

Figure S5 Sequence saturation analysis of eight samples. The X-axis shows the number of clean reads, units is 100 k, the Y-axis shows the number of genes, units is %.

Figure S6 Scatter plot of top 20 KEGG pathways of two pairwise comparisons (GD/GS, PD/PS).

Table S1 Summary for the transcriptome sequencing and assembly.

Table S2 Summary of EST-SSR searching results.

Table S3 Gene-specific primers used in quantitative real-time PCR.

Table S4 Differentially expressed genes between GD and GS.

Table S5 Differentially expressed genes between PD and PS.
