# Supplementary figures and images for: Transcriptome and Differential Expression Profiling Analysis of the Mechanism of Ca2+ Regulation in Peanut (Arachis hypogaea) Pod Development
Source: Front Plant Sci. 2017 Sep 28;8:1609. doi: 10.3389/fpls.2017.01609 (PMC5625282; doi:10.3389/fpls.2017.01609)

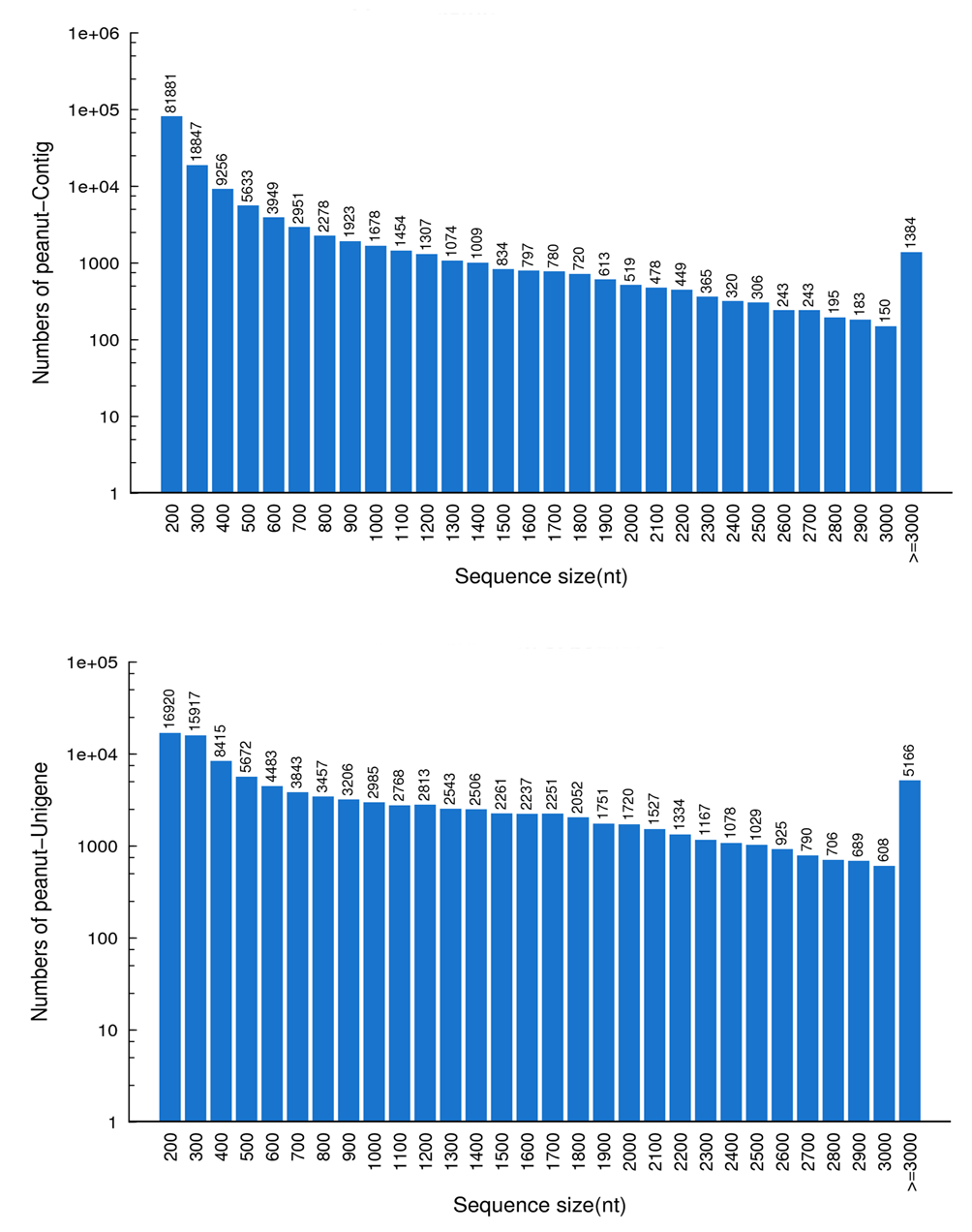

Supplement: Figure S1 — The length distribution of contigs and unigenes. [file Image1.TIF]

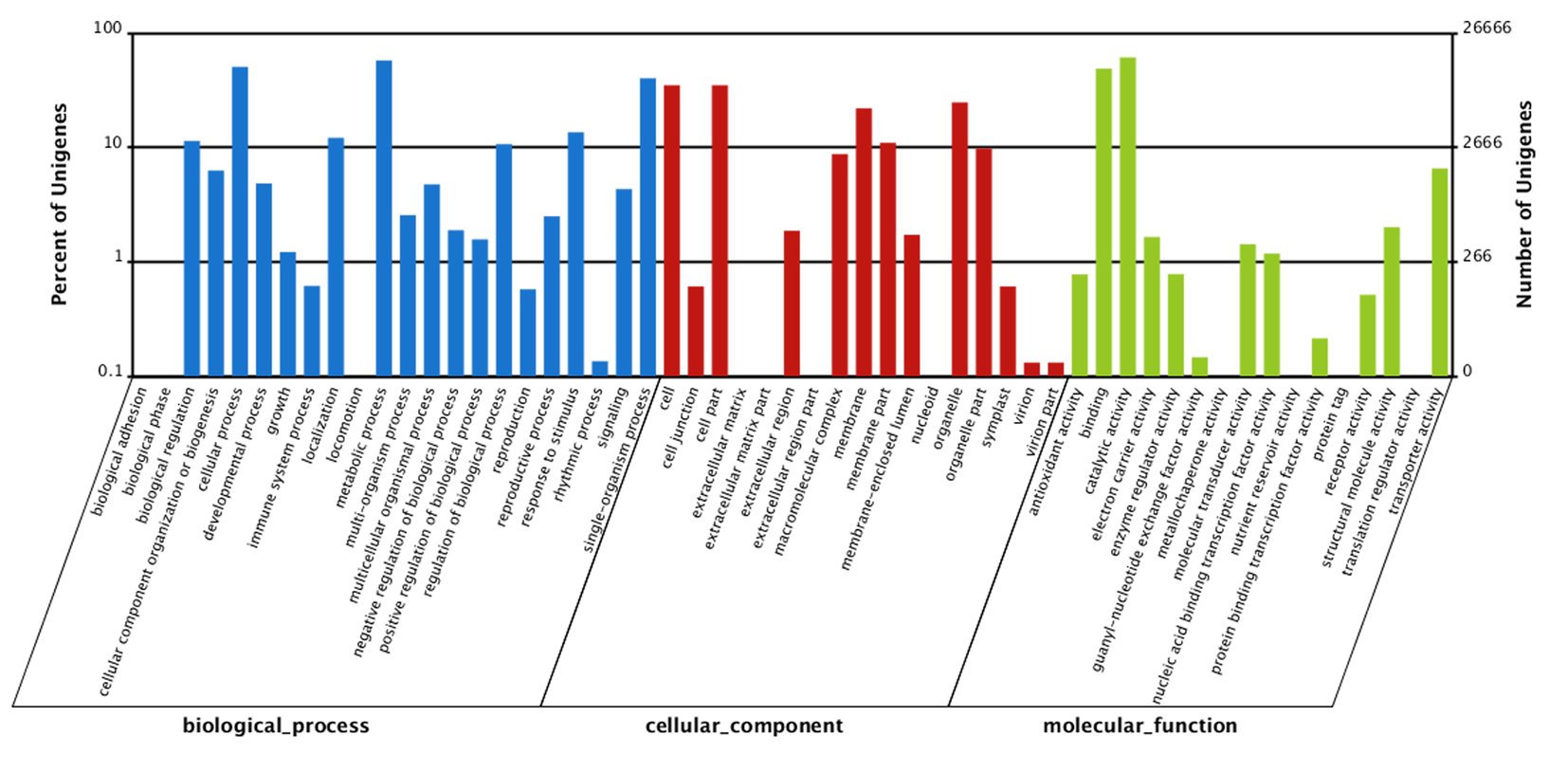

Supplement: Figure S2 — GO classification analysis of unigenes in All-unigene. GO functions is showed in X-axis. The right Y-axis shows the number of genes which have the GO function, and the left Y-axis shows the percentage. [file Image2.TIF]

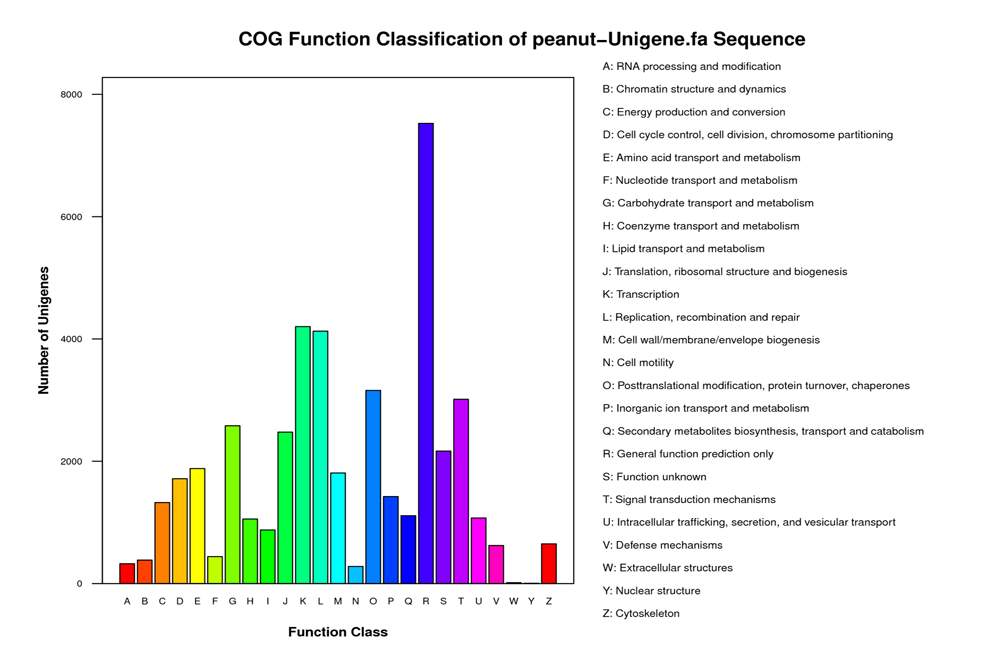

Supplement: Figure S3 — Clusters of orthologous groups (COG) classification of Unigenes in All-Unigene. [file Image3.TIF]

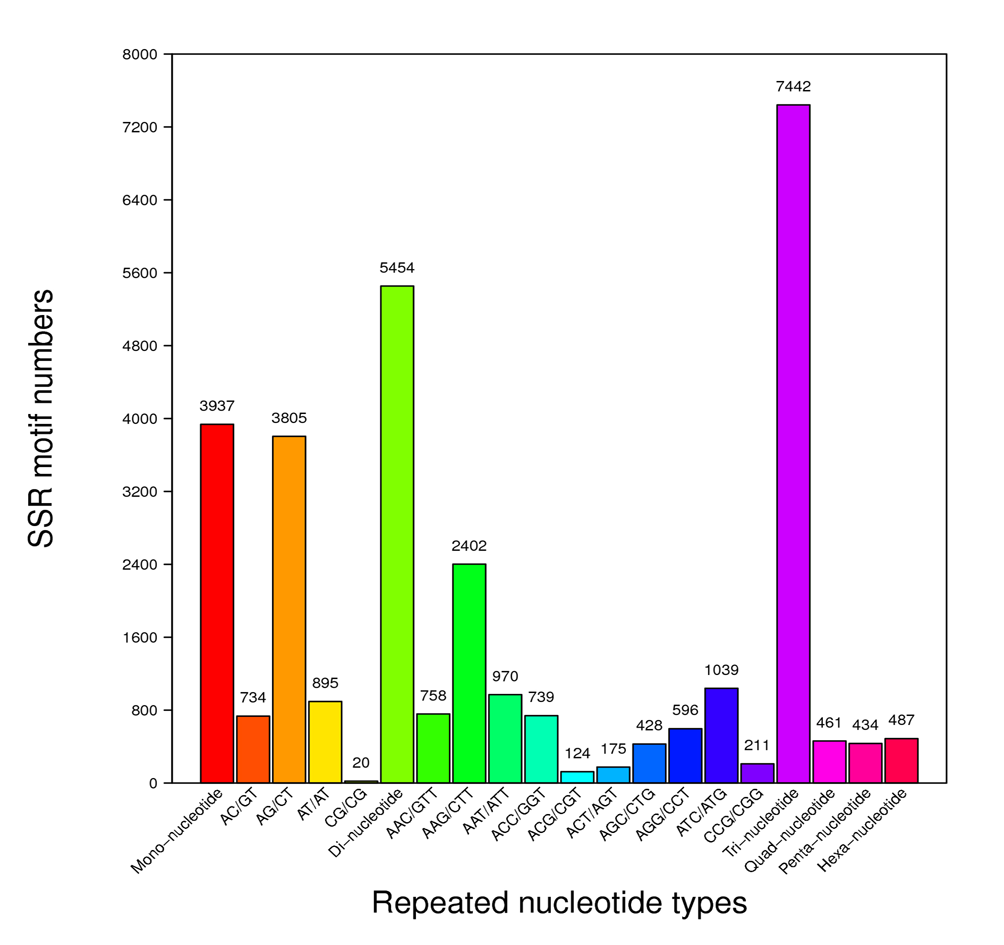

Supplement: Figure S4 — Quantity statistics of SSR classification. The X-axis is the repeat times of repeat units. The Y-axis is the number of SSRs. [file Image4.TIF]

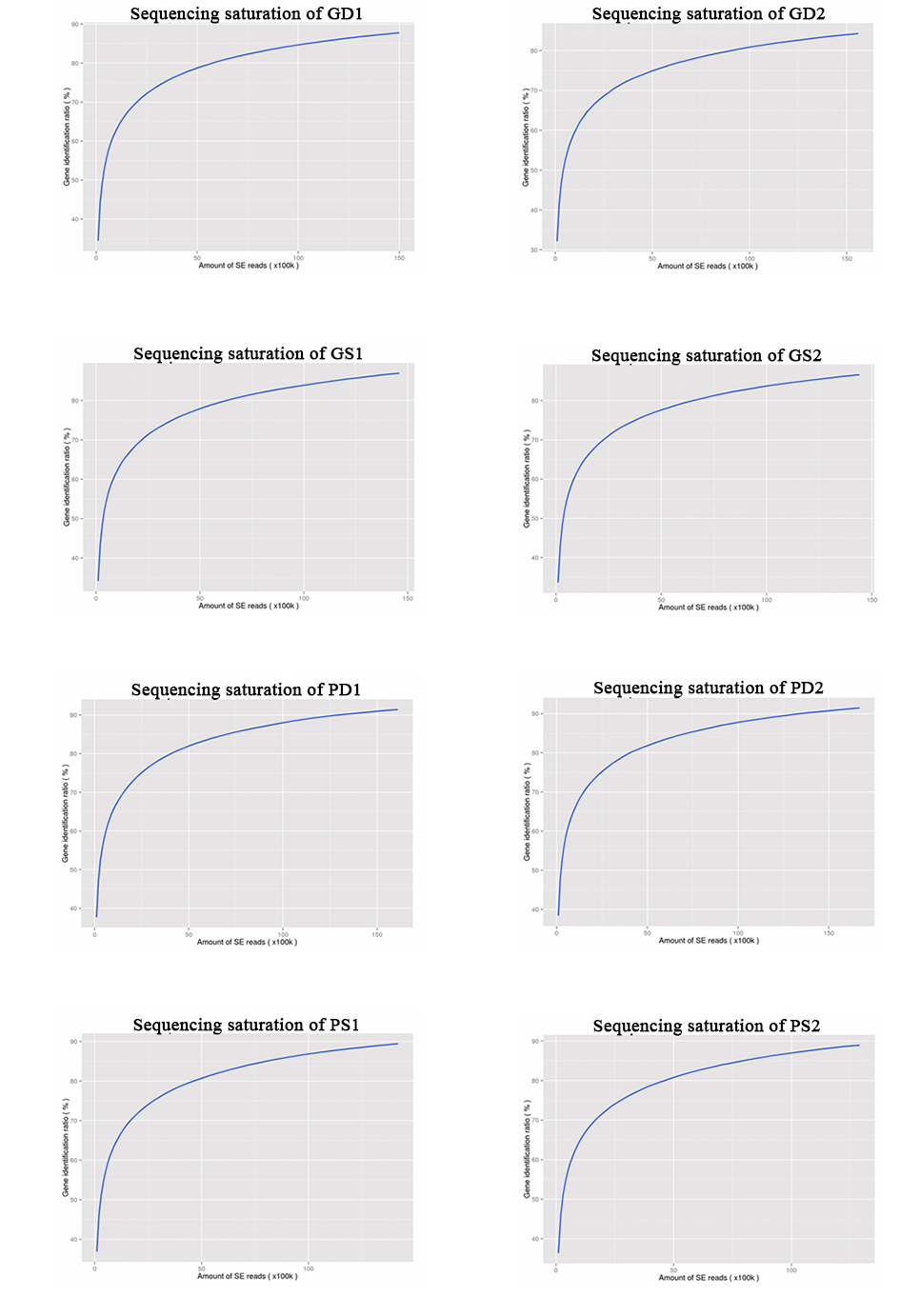

Supplement: Figure S5 — Sequence saturation analysis of eight samples. The X-axis shows the number of clean reads, units is 100 k, the Y-axis shows the number of genes, units is %. [file Image5.TIF]

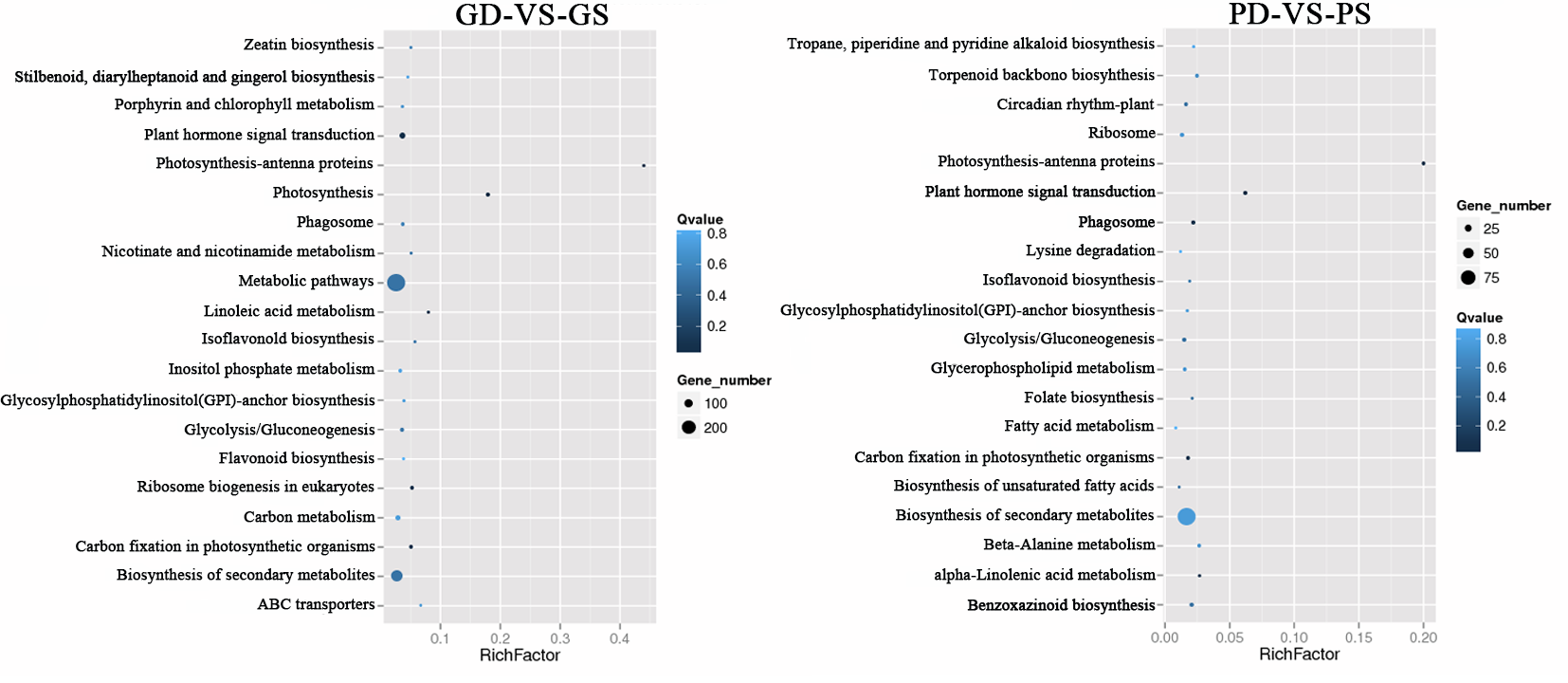

Supplement: Figure S6 — Scatter plot of top 20 KEGG pathways of two pairwise comparisons (GD/GS, PD/PS). [file Image6.TIF]
