# Supplementary material for: Transcriptome and Differential Expression Profiling Analysis of the Mechanism of Ca2+ Regulation in Peanut (Arachis hypogaea) Pod Development
Source: Front Plant Sci. 2017 Sep 28;8:1609. doi: 10.3389/fpls.2017.01609 (PMC5625282; doi:10.3389/fpls.2017.01609)
Supplement: Table S1 — Summary for the transcriptome sequencing and assembly. [file Table1.DOCX]

**Table S1** Summary for the transcriptome sequencing and assembly.

| Total number of reads |  |
| --- | --- |
| Total raw reads | 100,654,030 |
| Total clean reads | 99,030,828 |
| Total clean nucleotides (nt) | 9,903,082,800 |
| Q20 percentage | 97.90% |
| N percentage | 0.00% |
| GC percentage | 44.97% |
| Total number of contigs | 141,819 |
| Total length of contigs (nt) | 55,502,183 |
| Mean length of contigs (nt) | 391 |
| N50 of contigs | 856 |
| Total number of unigenes | 102,819 |
| Total length of unigenes (nt) | 102,767,513 |
| Mean length of unigenes (nt) | 999 |
| N50 of unigenes | 1782 |
| Total consensus sequences | 102,819 |
| Distinct clusters | 46,239 |
| Distinct singletons | 56,580 |
