# Supplementary material for: Transcriptome and Differential Expression Profiling Analysis of the Mechanism of Ca2+ Regulation in Peanut (Arachis hypogaea) Pod Development
Source: Front Plant Sci. 2017 Sep 28;8:1609. doi: 10.3389/fpls.2017.01609 (PMC5625282; doi:10.3389/fpls.2017.01609)
Supplement: Table S2 — Summary of EST-SSR searching results. [file Table2.DOCX]

**Table S2** Summary of EST-SSR searching results.

| Searching item | Numbers |
| --- | --- |
| Total number of sequences examined | 120,819 |
| Total size of examined sequences (bp) | 102,767,513 |
| Total number of identified SSRs | 18215 |
| Number of SSR containing sequences | 14985 |
| Number of sequences containing more than 1 SSR | 2493 |
| Number of SSRs present in compound formation | 974 |
| Mono-nucleotide | 3937 |
| Di-nucleotide | 5454 |
| Tri-nucleotide | 7442 |
| quad-nucleotide | 461 |
| Penta-nucleotide | 434 |
| Hexa-nucleotide | 487 |
