# Supplementary material for: Transcriptome and Differential Expression Profiling Analysis of the Mechanism of Ca2+ Regulation in Peanut (Arachis hypogaea) Pod Development
Source: Front Plant Sci. 2017 Sep 28;8:1609. doi: 10.3389/fpls.2017.01609 (PMC5625282; doi:10.3389/fpls.2017.01609)
Supplement: Table S3 — Gene-specific primers used in quantitative real-time PCR. [file Table3.DOCX]

| Gene Name | Primer | Primer Sequence (5’→3’) |
| --- | --- | --- |
| Unigene14989 | F | GATATTAGACCACTCTGATC |
|  | R | TCCTCATCATCCTACTAACAATG |
| CL6636.Contig4 | F | CAGGCAGCTTAACACATAC |
|  | R | GAGATGGTTGCTGAGTACTAC |
| CL1486.Contig8 | F | GTGAAGAGGATGAGGACG |
|  | R | TCCATCAGCGCTGCCTC |
| CL8267.Contig2 | F | AAGGTGAAGTCTATGCATG |
|  | R | TGTGTTTGAGCTTTGTGG |
| CL790.Contig7 | F | TAAGAATCAGGGGTTACAG |
|  | R | GGTAACTGTCCAAGTGAAG |
| Unigene31203 | F | CCAATATGAGAGGTGTGGTTC |
|  | R | ACACAAAAGATAACACTGAGAG |
| CL1022.Contig2 | F | CACTAGGACTAGCACTACT |
|  | R | GACATGTGATTAGAGCTTG |
| Unigene12500 | F | GAACCAAACCTACATGCAT |
|  | R | ATGATCTTGTAGGTAGCGGT |
| Unigene23103 | F | TCTACCGCCGCTCCAAAAC |
|  | R | TCGATGGCAGGGTCATGAG |
| CL12322.Contig1 | F | ACAATGGCAGATATTCTTAGTG |
|  | R | AGCTCTTCTTCAGTTGGGTTC |

**Table S3** Gene-specific primers used in quantitative real-time PCR.
